# Wzi Is an Outer Membrane Lectin that Underpins Group 1 Capsule Assembly in *Escherichia coli*

**DOI:** 10.1016/j.str.2013.03.010

**Published:** 2013-05-07

**Authors:** Simon R. Bushell, Iain L. Mainprize, Martin A. Wear, Hubing Lou, Chris Whitfield, James H. Naismith

**Affiliations:** 1 Biomedical Sciences Research Complex, University of St Andrews, St Andrews, KY16 9ST, UK; 2 Department of Molecular & Cellular Biology, University of Guelph, Guelph, Ontario N1G 2W1, Canada; 3 School of Chemistry, King’s Buildings, University of Edinburgh, Edinburgh, EH9 3JJ, UK

## Abstract

Many pathogenic bacteria encase themselves in a polysaccharide capsule that provides a barrier to the physical and immunological challenges of the host. The mechanism by which the capsule assembles around the bacterial cell is unknown. Wzi, an integral outer-membrane protein from *Escherichia coli,* has been implicated in the formation of group 1 capsules. The 2.6 Å resolution structure of Wzi reveals an 18-stranded β-barrel fold with a novel arrangement of long extracellular loops that blocks the extracellular entrance and a helical bundle that plugs the periplasmic end. Mutagenesis shows that specific extracellular loops are required for in vivo capsule assembly. The data show that Wzi binds the K30 carbohydrate polymer and, crucially, that mutants functionally deficient in vivo show no binding to K30 polymer in vitro. We conclude that Wzi is a novel outer-membrane lectin that assists in the formation of the bacterial capsule via direct interaction with capsular polysaccharides.

## Highlights

• Wzi is an 18-stranded β-barrel outer-membrane protein
• Wzi has a unique N-terminal helical domain and novel extracellular loops
• In vivo data show that extracellular loops are required for capsule formation
• In vitro data show that these loops are required to bind K30 polymer

## Teaser

Wzi is a novel β-barrel outer membrane protein that is found in bacteria that make group 1 capsules. When the protein is missing or specific regions are mutated, capsule formation is impaired. Bushell et al. show that the protein binds to K30 capsular polysaccharide and suggest it templates capsule assembly.

## Introduction

Many species of bacteria, including both Gram-positive and Gram-negative organisms, are enveloped by a capsule. Capsules are comprised of high-molecular-weight capsular polysaccharide (CPS) chains that are exported across the bacterial cell envelope and retained at the cell surface. The capsule provides protection from environmental stress, including the immune response of the infected host (Roberts, 1996). The chemical composition of CPS displays remarkable diversity even within a single species; *Escherichia coli* has over 80 different capsular (K antigen) serotypes, for example (Jann and Jann, 1987; Orskov et al., 1977). *E. coli* capsules have been subdivided into four groups based on structural, biochemical, and genetic criteria, and these identify model systems applicable to capsules from across the Bacteria kingdom (reviewed in Whitfield, 2006). CPS chains in groups 2 and 3 are thought to be linked to the surface via a diacylglycerolphosphate moiety (Gotschlich et al., 1981), although it was recently proposed that lipopolysaccharide (LPS) structures influence CPS association (Jiménez et al., 2012). In groups 1 and 4, shorter carbohydrate chains are incorporated into LPS molecules with lipid A as the membrane anchor (Amor and Whitfield, 1997; MacLachlan et al., 1993). Longer chains assemble to form capsules independently of covalent attachment to LPS. The mechanism by which group 1 CPS chains are assembled into a coherent cell-associated capsule structure rather than being secreted as exopolysaccharides (EPSs) has yet to be elucidated. The controlled assembly of polar polymers into well-defined three-dimensional structures is of interest in chemistry and biology.

The K30 capsule represents the prototype for group 1 capsules (related to group 4) (Whitfield, 2006). The K30 antigen is comprised of repeat units containing a [-2)-α-Man-(1-3)-β-Gal-(1-] backbone with a disaccharide branch of β-GlcA-(1-3)-α-Gal attached to the 3-position of the main-chain mannose (Chakraborty et al., 1980). The polymer also has nonstoichiometric O-acetylation at Gal residues (Steiner et al., 2007). Its synthesis begins in the cytoplasm, where membrane-associated glycosyltransferases assemble an undecaprenyl diphosphate (und-PP)-linked K30 tetrasaccharide repeat unit (Whitfield, 2006). Once assembled, the integral membrane flippase Wzx exports the lipid-linked K30 repeat unit to the periplasmic face of the inner membrane, where it is extended by the Wzy polymerase (Drummelsmith and Whitfield, 1999). Higher-order polymerization of the CPS is regulated by the tyrosine autokinase, Wzc, a member of the Polysaccharide CoPolymerase (PCP-2a) family. This protein forms an oligomer (Collins et al., 2007), with the larger periplasmic domain composed of helical bundles provided by each monomer (Cuthbertson et al., 2009; Morona et al., 2009). The cytoplasmic domain of PCP-2a proteins contains autokinases that can oligomerize to form an octameric ring structure, which is disrupted when phosphorylated (Olivares-Illana et al., 2008). The cycling of the cytoplasmic phosphorylation state of Wzc is crucial to export and is regulated by its cognate phosphatase, Wzb (Hagelueken et al., 2009a; Nadler et al., 2012; Vincent et al., 1999; Wugeditsch et al., 2001). Wza is the outer-membrane export channel (Nesper et al., 2003). Wza has a large central cavity that is thought to mediate passage of the polymer across the outer membrane (Dong et al., 2006a). Although the purified Wza octamer is closed in both crystal and solution (Hagelueken et al., 2009b), it must open to permit passage of CPS. The periplasmic domains of Wza and Wzc interact to form a transperiplasmic complex that may facilitate opening (Collins et al., 2007).

Like other members of the Wzy-dependent pathway, the outer-membrane protein Wzi is encoded by the *cps* gene cluster. Group 1 capsule-producing strains of *E. coli* secrete CPS into the extracellular environment when the *wzi* gene is knocked out, with the remaining cell-associated CPS unable to form a coherent capsule layer (Rahn et al., 2003). *Klebsiella pneumoniae* possesses an identical group 1 capsule assembly system (Rahn et al., 1999) with *wzi* knockouts similarly showing a reduction in cell-associated capsule (Alvarez et al., 2000). Wzi homologs are absent in organisms that use a Wzy-dependent pathway to synthesize secreted EPSs, e.g., colanic acid EPSs in *E. coli* (Rahn et al., 1999; Reid and Whitfield, 2005), the amylovoran and stewartan biosynthetic pathways in *Erwinia* spp. (Bugert and Geider, 1995; Carlier et al., 2009).

The crystal structure of Wzi reveals an 18-stranded β-barrel with a striking periplasmic helical bundle. Biophysical data demonstrate that Wzi specifically recognizes the capsular polysaccharide. Loss of carbohydrate binding leads to loss of function in vivo. We hypothesize that the Wzi-polysaccharide interaction is the critical initialization step in the organization of functional capsule.

## Results

### Wzi Is a β-barrel

**Figure 1:**
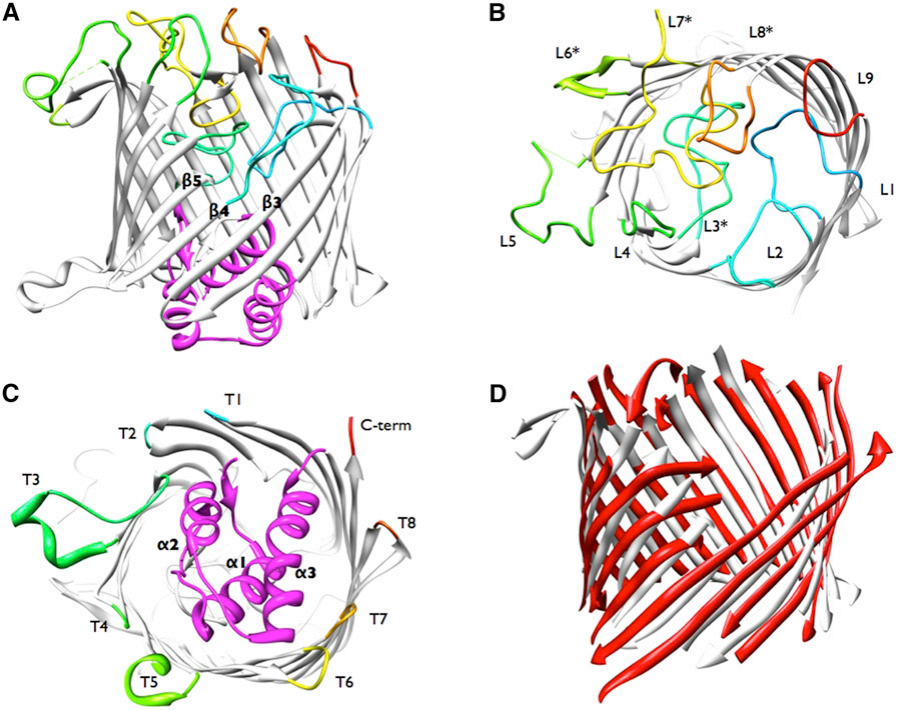
Crystal Structure of Wzi (A) The X-ray crystal structure of Wzi reveals a β-barrel fold. Wzi is oriented with the extracellular face at the top. The extracellular loops are colored from red to blue according to primary sequence, and the periplasmic N-terminal helical bundle is colored magenta. Sheets β3, β4, and β5 are labeled. (B) Top view of extracellular loops gradient colored according to primary sequence. Loops with asterisks indicate the loops that were deleted for assay; the helical bundle has been removed for clarity. (C) Bottom view of periplasmic turns, gradient colored according to primary sequence. (D) Structural alignment of Wzi (white) with its closest structural homolog PflBenF, a BenF-like outer-membrane protein from *Pseudomonas fluorescens* (red, PDB ID code 3JTY) (Sampathkumar et al., 2010). The alignment was generated using PDBeFold (Krissinel and Henrick, 2004) and extracellular loops and helices have been removed for clarity. Structural figures were created with UCSF Chimera (Pettersen et al., 2004). See also Figures S1, S2, and S4.

The X-ray structure reveals Wzi to be an 18-stranded antiparallel β-barrel, with the wall of the barrel describing a circle approximately 36 Å in diameter (Figures 1A and 1B). This differs from the elliptical arrangement seen in β-barrel channel-forming porins such as maltoporin (Schirmer et al., 1995). Visual inspection and analysis with PISA (Krissinel and Henrick, 2007) indicates that Wzi is a monomer. The first-ordered residue is A24 (residues 1–23 are the cleaved signal peptide) and the last-ordered residue is L477 (C terminus of the mature protein). Not seen are residues 275–287, which form part of an extended loop between β9 and β10 (loop L5) and the C-terminal His tag. The barrel has a striking α-helical bundle (α1-3) that precedes the N-terminus of the first strand (Figures 1A, 1C, and Figure S1 available online). Since the helical region, the N-terminus of the first strand, and the C terminus are all at the same end of the barrel, we concluded that the helices are periplasmic (confirmed experimentally below). The faces of the Wzi barrel, which are exposed at the interior and exterior surfaces of the outer membrane, are notably asymmetric. For clarity, we designate the extracellular strand connections as loops L1–L9, and periplasmic strand connections as “turns” numbered T1–T8. Extensive loops are found on the extracellular end of the barrel (Figures 1A and 1B), with the helical bundle dominating the periplasmic face folding into the barrel (Figures 1A and 1C). The long extracellular loops—L3, L5, and L7—adopt distinct structures, parts of which fold into the barrel, whereas loops L5, L6, and L7 extend beyond the circumference of the barrel wall (Figure 1B). Loop L6 has several exposed hydrophobic residues, suggesting that these residues are inserted into the membrane. The other loops are folded into the barrel and completely occlude the pore (PoreWalker [Pellegrini-Calace et al., 2009]). At the other end of the barrel, the protein is fixed into the membrane by exposed aromatic residues likened to a hydrophobic “belt”: a common feature in polytopic membrane proteins (Wolfenden, 2007). Turns T3 and T5 are more extensive, splaying outward from the barrel, and contain a short helical region with exposed tryptophans. The lengths of the β strands are irregular; β4 is only six residues long and results in a triangular “notch” at the extracellular face of the barrel.

The closest homolog found by secondary structure matching (SSM) is a monomeric BenF-like protein of unknown function (Protein Data Bank [PDB] ID code 3JTY [Sampathkumar et al., 2010]) from *Pseudomonas fluorescens* (root-mean-square deviation [rmsd], 2.79 Å; *Z* score, 8.1) (Figure 1D). Like Wzi, PflBenF is a monomer with a circular arrangement of strands. PflBenF shares only a 14% sequence identity (31% similarity) with Wzi. Further, PflBenF has no N-terminal helices and has generally shorter periplasmic loops, with two exceptions: one is polar and points into the periplasm, whereas the other is hydrophobic and splays outward from the barrel, similar to T3 in Wzi. Some of the loops on the extracellular face of PflBenF fold into the central cavity, but not to the extent observed for Wzi. In addition, the extracellular loops of PflBenF constrict rather than occlude the pore. PflBenF bears more similarity to porins such as OmpC (Baslé et al., 2006; Tamber et al., 2006) than to Wzi.

LamB is an exemplar of the 18-stranded trimeric barrel structures (Schirmer et al., 1995). LamB is very different from Wzi, lacking the N-terminal helices and the extensive extracellular loops. LamB has an elliptically shaped barrel wall that prevents a meaningful superposition with Wzi. LamB also has a clear central channel (Schirmer et al., 1995).

The helices of the triple α-helical bundle (α1–3) of Wzi are amphipathic, with their hydrophobic faces stacked against each other. *PDBeFold* (Krissinel and Henrick, 2004) identifies a similar arrangement of helices in DnaJ-like domains. These domains are seen in a variety of cochaperone proteins across all kingdoms of life, although other functions have been assigned to these proteins (Walsh et al., 2004). The helical bundle of Wzi shares its highest structural similarity (rmsd = 2.15 Å; *Z* score = 3.8) with Tim16, a DnaJ-like protein involved in the posttranslational import of proteins into yeast mitochondria (Mokranjac et al., 2006) (Figure S2B). The functional significance of this structural similarity is unclear, as Wzi lacks other characteristic sequence motifs that have been used to define the J-domain.

### Orientation of Wzi-FLAG in the Membrane

The N-terminus of the first strand of a β-barrel is characteristically periplasmic (Schulz, 2000). Due to the presence of the novel helical bundle, the orientation of the Wzi barrel in the outer membrane was confirmed experimentally using a C-terminal epitope-tagged Wzi-FLAG derivative expressed in *E. coli* TOP10 cells. The data indicate that the FLAG epitope was not exposed to antibodies on the surface of intact cells (Figure S2A). However, a robust fluorescent signal was observed after the cells were permeabilized, with anti-FLAG antibodies able to access and bind the epitope at the C terminus (Figure S2A). This confirms that Wzi is oriented such that its C terminus and, by extension, its N-terminal helical bundle are both exposed to the periplasm.

### Characterization of the Phenotypes of Wzi Loop-Deletion Mutants

**Figure 2:**
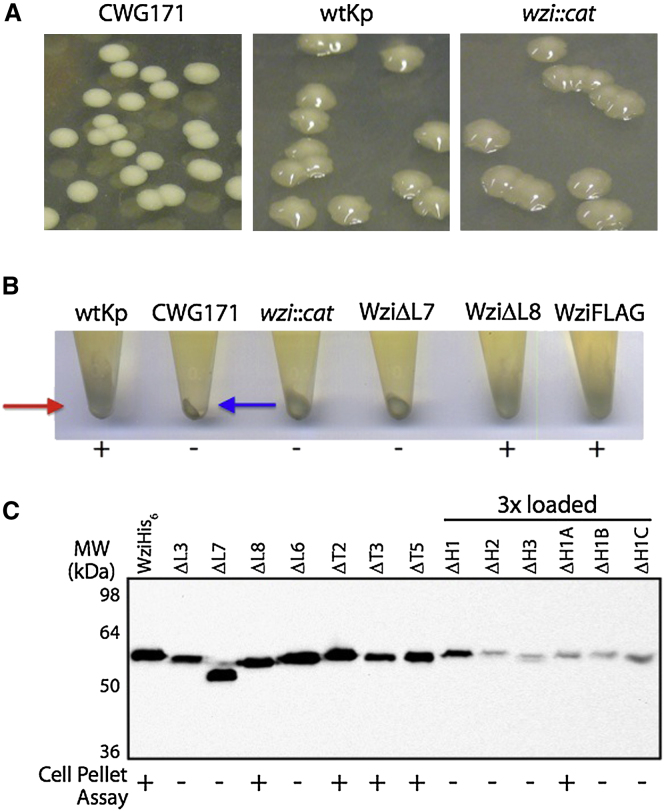
Analysis of Wzi Mutants (A) Colonies of wild-type *K. pneumoniae* 889/50 (wtKp, serotype O1:K20) and CWG874 (*wzi*::*cat*) were indistinguishable on LB agar plates but were different from those of a capsule-deficient strain, CWG171 (*K. pneumoniae* O1:K^−^, strain KD2 (McCallum et al., 1989)). (B) After centrifugation, wild-type *K. pneumoniae* 889/50 formed a diffuse pellet (red arrow), indicative of a coherent capsule. The pellet from CWG874 was more compact, similar to the pellet from the unencapsulated CWG171 (blue arrow). Shown here as examples, expression of WziΔL8 and WziFLAG, but not WziΔL7, could produce a cell pellet phenotype identical to that of the wild-type strain. (C) Immunoblotting of cell lysates of CWG874 expressing Wzi mutants probed with anti-His_5_ antibodies (QIAGEN). Three times more sample was loaded for the helix-deletion mutants relative to the other samples, due to reduced expression levels. The blot was further contrast enhanced to visualize the fainter bands of the helix mutants. A summary of the cell pelleting results for each Wzi mutant is listed below the blot; “+” represents a rescue of the wild-type cell pellet phenotype. See also Tables S1 and S2.

*Klebsiella pneumoniae* 889/50 produces a serotype K20 capsule that is chemically identical to that of *E. coli* K30 (Choy and Dutton, 1972). The corresponding genetic loci are highly conserved, which is indicative of lateral gene transfer (Rahn et al., 1999). Colonies of *K. pneumoniae* 889/50 are much more mucoid than their *E. coli* counterparts due to their production of large amounts of CPS. We reasoned that this property would be beneficial in analyzing in vivo capsule biosynthesis in Wzi mutants (Figure 2A). High-level CPS production represents a challenge in harvesting *K. pneumoniae* cells by centrifugation and can be exploited in a qualitative assay for encapsulation. The same approach has been used in other studies (Lai et al., 2003; Wu et al., 2011). Wild-type *K. pneumoniae* 889/50 formed a loose, wispy cell pellet after low-speed centrifugation (Figure 2B). Under the same conditions, unencapsulated mutants (e.g., CWG171, Figure 2B) form small, compact pellets. Despite the mucoid morphology of colonies on solid media, which are indistinguishable from the wild-type (Figure 2A), CWG874 (*wzi*::*cat*) behaved more like an unencapsulated strain after centrifugation. The wild-type phenotype was rescued by expression of plasmid-encoded Wzi in *trans*. This centrifugation assay was used to probe mutants of Wzi to identify regions important for function.

Loops L8 (completely surface-exposed), L7 (partially buried), L3 (completely buried), and L6 (extending out perpendicular to the barrel) (Figures 1B and S1) were targeted for deletion. These were selected because their extensive structural features made them likely to have a functional role. Furthermore, L6–L8 cluster together to form a positive electrostatic patch, which suggests that they interact with the negatively charged sugar. We also deleted the two longest turns, T3 and T5, which extend perpendicular to the barrel at the periplasmic face, as their structure and length offer the potential to be more than just a means of connecting adjacent β-stands (Figure 1C). One of the shorter turns, T2, was deleted as a control. The α-helical bundle, which distinguishes Wzi from other β-barrel structures, was probed; individual helices were deleted and short amino acid stretches of helix H1 were substituted with glycines.

Of the four loops deleted on the extracellular face of Wzi (loops L3 and L6–L8), only loop L8 was expendable (Figures 2B and 2C). Deletion of the three periplasmic turns (T2, T3, and T5) had no detectable effect on activity and, like the loop mutants, they were expressed at robust levels (Figure 2C). The N-terminal helices were all essential for function, but expression levels for these helical mutants, particularly ΔH1 and ΔH2, were low (Figure 2C). To rule out the possibility that the null phenotype was solely due to low expression, more conservative approaches were used. Substitution mutagenesis of the first five amino acids of H1 (residues 32–36, ΔH1A) by glycine had no effect on Wzi function, although the mutant Wzi was still expressed at a low level. Replacement of the central five (35–39) or last five (39–43) residues was deleterious for function. The observation that Wzi-ΔH1A is functional in vivo suggests that either the extensive hydrogen-bonding network seen between the side chain of R34 of helix H1 and P177/G178 from loop L3 is not essential for function or the structure reorganizes to preserve key interactions.

### In Vitro Characterization

**Figure 3:**
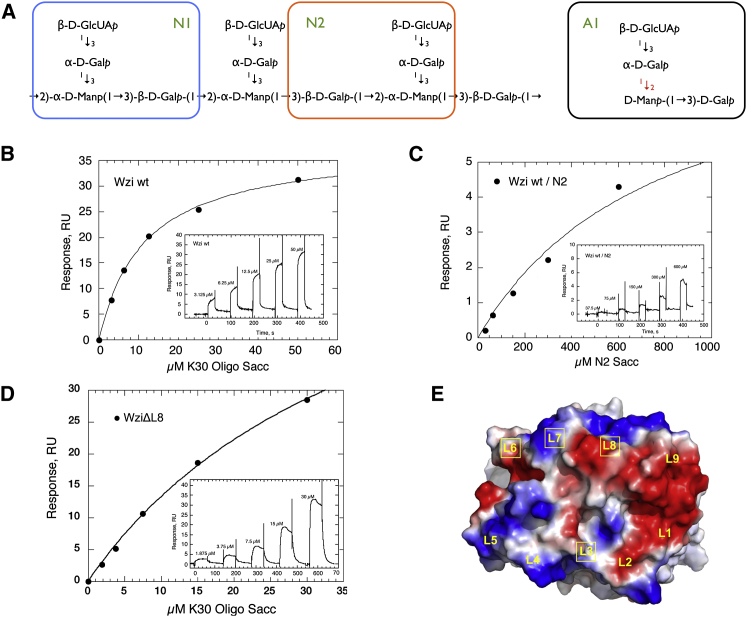
Wzi Binds to K30-Derived Oligosaccharides (A) Segments of the K30 CPS repeat unit, comprising two different tetrasaccharides (N1 and N2), were tested for binding. A third analogous tetrasaccharide (A1, right*)* was also tested. (B) Steady-state affinity curve of immobilized Wzi for polymeric K30 measured by SPR. (Inset) SPR sensorgram of K30 concentration series titrated over Ni-NTA-immobilized Wzi. (C) Steady-state affinity curve of immobilized Wzi for monomeric K30N2. (Inset) SPR sensorgram of a K30N2 concentration series titrated over Ni-NTA-immobilized Wzi. (D) Steady-state affinity curve of immobilized WziΔL8 for K30 polymer. (Inset) SPR sensorgram of K30 polymer concentration series titrated over Ni-NTA-immobilized WziΔL8. (E) Vacuum electrostatic rendering of the extracellular surface of Wzi. The location of each extracellular loop is labeled. Boxed loops indicate the loops that were deleted and tested for binding to K30. Figure constructed using the PyMOL Molecular Graphics System (Version 1.5.0.3, Schrödinger, LLC). See also Table S1 and Figures S3 and S5.

Surface plasmon resonance (SPR) was used to probe the affinity of Wzi for the K30 CPS (Figure 3A). When a solution of K30 was titrated over immobilized Wzi (Figure 3B), a reproducible binding curve was observed, indicating a specific interaction between Wzi and polymeric K30 CPS. The response was lower than predicted based on the expected sizes of components, suggesting that the specific activity of the immobilized protein is low (possibly due to the manner in which the protein binds to the chip). Extracted K30 CPS has an average apparent molecular weight of 100–150 kDa, judged by mobility on an SDS-PAGE gel (Hungerer et al., 1967), and this heterogeneity precludes reliable calculation of a dissociation constant (*K*_*d*_). However, an estimate of 100 kDa yields an estimated *K*_*d*_ of 11.5 ± 0.7 μM, a value that, we stress, is qualitative. The binding experiments were repeated using three tetrasaccharides (Figure 3A). Two of the molecules (N1 and N2) are contained within the K30 repeat, whereas the third (A1) is closely related to the polysaccharide building block. Only K30N2, showed any binding signal, with an interaction weaker than polymer (*K*_*d*_ = ∼800 μM) (Figure 3C).

WziΔL3, WziΔL6, WziΔL7, and WziΔL8 were also tested for their capacity to bind polymeric K30. WziΔL8 retained binding with a slightly weaker *K*_*d*_ of 41 ± 4 μM (Figure 3D) compared to native. WziΔL3, WziΔL6, and WziΔL7 showed no detectable binding (Figure S3).

## Discussion

Eighteen-stranded β-barrel structures are well known (Biswas et al., 2007; Fairman et al., 2011), but they adopt a distinctive elliptical shape. One 18-stranded β-barrel, BenF from *P. fluorescens* (Sampathkumar et al., 2010), has the circular arrangement of Wzi but lacks its extensive extracellular loops and helical bundle. Wzi differs fundamentally from the porin class of membrane proteins in that it is sealed at both ends and only an unprecedented rearrangement could create a pore. Many β-barrel proteins contain functional N-terminal domains. For example, the ferric enterobactin receptor (FepA) (Buchanan et al., 1999) and closely related TonB-dependent receptors possess an N-terminal “cork” domain that sits in the center of the barrel, occluding the pore. The cork is thought to move aside to permit substrate import. In BtuB (and relatives), the N-terminal domain provides a scaffold for protein-protein interactions that couples to the TonB system (Kurisu et al., 2003; Shultis et al., 2006), a multiprotein periplasmic assembly that provides energy for substrate uptake (Noinaj et al., 2010). The arrangement of helices in Wzi is distinct from either of these families and lacks the TonB recognition signal. FadL (and TodX) has an N-terminal helical bundle (van den Berg et al., 2004) but with a different topology.

Some integral outer-membrane β-barrels feature a shortened β sheet that creates a “notch” (or crenel) at the extracellular edge of the structure that may bind to ligands, or possibly other proteins. For example, it has been proposed that FadL, a fatty acid transporter, uses this mechanism to release fatty acids laterally into the membrane (van den Berg et al., 2004). PagP, an LPS lipid A acyltransferase, also uses a notch motif to bind its phospholipid donor (Khan and Bishop, 2009). Wzi has a notch region, but it is smaller than the corresponding regions in Fad or PagP (Figure S4), and Wzi lacks a central cavity. In summary, Wzi has no existing structural paralog.

**Figure 4:**
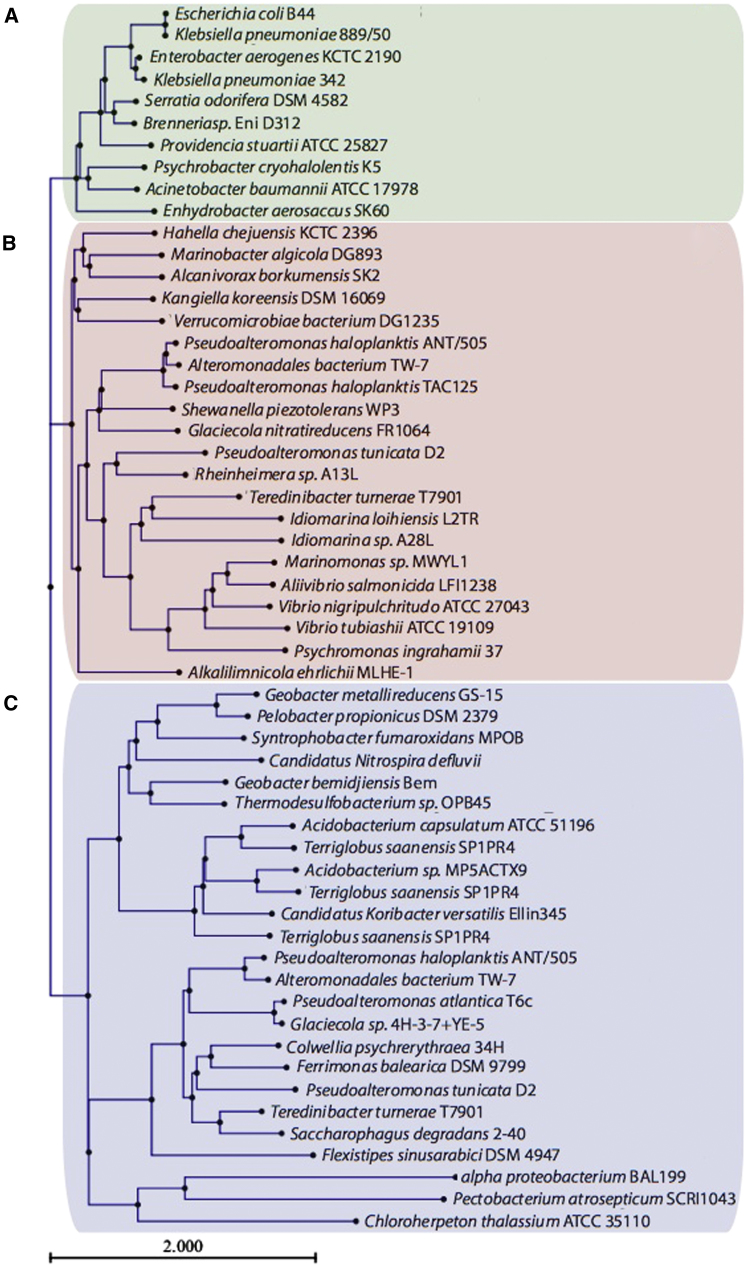
Phylogenetic Tree of Wzi Homologs The Wzi hits, identified by BLAST searches of completed bacterial genomes (12-2011) and refined by secondary-structure prediction, clustered into groups A–C. The Wzi hits in Groups A and B are all from organisms belonging to the Gammaproteobacteria class, except for *Verrucomicrobiae bacterium* DG1235, from the Verrucomicrobia phylum. Group C is more diverse, containing organisms from the following phyla: Acidobacteria, Nitrospirae, Thermodesulfobacteria, Deferribacteres, and Chlorobi, as well as many from Proteobacteria (Alpha, Delta, and Gamma classes). This tree has been abbreviated for clarity; branches with multiple hits of the same genus were represented by the top hit of that branch, and bootstrap values were removed. See also Table S4.

Wzi proteins are found in the prototype organisms for group 1 (Wzy-dependent) capsules, *E. coli* and *K. pneumoniae*. There are relatively few Wzi sequence homologs in other organisms. The majority of these species belong to the *Proteobacteria* phylum, but some are found in more diverse phyla (see Figure 4). Establishing a definitive shared role for the potential Wzi homologs across bacteria is difficult due to the limited knowledge regarding the capsular status of many of these organisms. However, we note that Wzi-containing organisms all possess homologs of the group 1 CPS outer-membrane translocon Wza (Dong et al., 2006a; Drummelsmith and Whitfield, 2000). As noted in the introduction, the colanic acid (Rahn et al., 1999; Reid and Whitfield, 2005) and amylovoran (Bugert and Geider, 1995; Carlier et al., 2009) secretion pathways, from *E. coli* and *Erwinia spp.*, respectively, differ from group 1 CPSs in only one feature: they lack a Wzi homolog (Reid and Whitfield, 2005). These polymers are mostly in a cell-free (secreted) EPS form, mirroring the *wzi* gene knockout experiments (Rahn et al., 2003) that disrupt (cell-associated) capsule formation in *E. coli* and *K. pneumoniae*. Structure-guided deletion mutagenesis of Wzi appears to eliminate functional significance of the periplasmic turns. Deletion of the N-terminal helices led to proteins with low expression levels and an abolition of activity. We attribute loss of function in these mutants to structural destabilization rather than to intrinsic low expression. Support for this comes from other mutations targeted to disrupt the interaction between helix H1 and the barrel. Despite its low level of expression, a polyglycine substitution on an exposed region of the helix had no effect on function (Figure 2). Individual deletions of three extracellular loops (L3, L6, and L7) led to a loss of activity but retained expression levels. Deletion of L8 showed no effect. Loops L3, L6, and L7 pack together to form a complex, positively charged electrostatic surface consistent with binding of the negatively charged sugar (Figure 3E). We conclude that this extracellular region is critical to the function of Wzi, but we do not exclude the involvement of other regions.

We had Wzi screened against the Mammalian Printed Microarray (v4.2) by the Consortium for Functional Glycomics. Wzi showed significant, but weak, binding to some human glycans (Table S1). Encouraged, we used SPR to probe carbohydrate binding to authentic CPS polymer in vitro. A measurable interaction was found between Wzi and K30 polymer that is comparable to the 3 to 4 μM association observed for the well characterized complex between the lectin ConA and its trimannose ligand (Mandal et al., 1994; Naismith and Field, 1996). Quantitation of the binding constant is difficult due to heterogeneity of the K30 polymer. In general, monovalent lectin polysaccharide interactions tend to be in the low-μM range (reviewed in Zeng et al., 2012), similar to what we observe with Wzi. Wzi demonstrated a preference for a polymeric substrate (i.e., K30 capsule) and selectivity (binds one of three closely related tetrasaccharides) (Figure 3C). These data establish that substrate recognition by Wzi extends beyond a monosaccharide-binding site. We have not yet obtained diffracting crystals for any cocomplex of Wzi. We note that carbohydrate polymers adopt higher-order structures that can create conformational epitopes not evident in their repeating units but detectable by SPR (MacKenzie and Jennings, 2003). The higher affinity of Wzi for the polymer may reflect specific recognition of a preferentially exposed carbohydrate epitope only present in the polymeric substrate. We did not observe any evidence for multivalent effects (Figure S5). It has been reported that ionic interactions between the core oligosaccharide region of LPS and CPS of *K. pneumoniae* are important for encapsulation (Fresno et al., 2006). Mutants lacking negatively charged residues in the LPS core were unable to form a capsule and thus were attenuated for virulence. However, changes in LPS could have had an indirect effect on other components in the capsule pathway.

**Figure 5:**
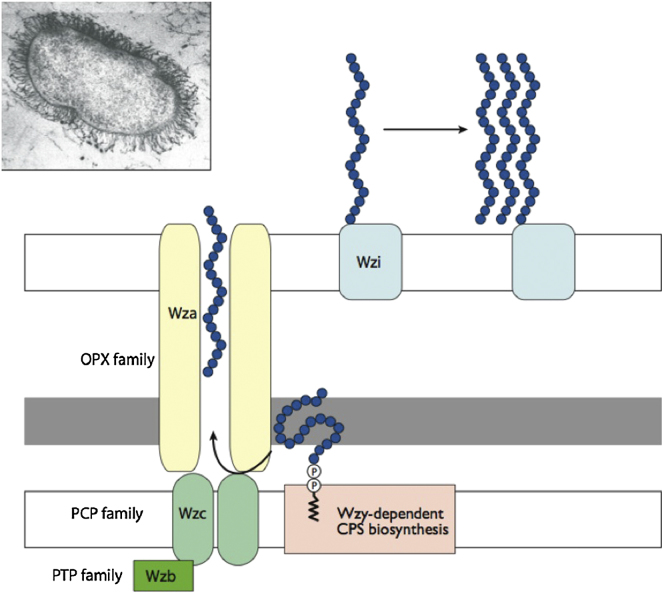
The Role of Wzi Lipid-linked components of the polysaccharide capsule are synthesized in the cytoplasm and extended by the Wzy-dependent pathway. Wza translocates polymerized capsular components en masse to the outer membrane. It is proposed that Wzi serves as an initial tethering point, capturing translocated polysaccharide that serves as a nucleation point for further secreted CPS. (Inset) *E. coli* with the K30 capsule.

We propose that Wzi exposed on the surface of the cell acts as a lectin to bind to nascent EPS as it leaves the cell through the Wza translocase. Essentially this creates a nucleation point that is a template for capsule formation (Figure 5). The organization of the *cps* cluster ensures the same level of transcription of genes encoding the major translocation/assembly proteins, *wzi* and *wza*, presumably to maintain a certain stoichiometry (Rahn and Whitfield, 2003). Given that Wza is active as an octamer (Dong et al., 2006b) and that the structure of Wzi indicates a monomeric state, the amount of functional Wzi should substantially exceed that of the essential export channel. In *E. coli*, trimeric porins represent the most abundant outer-membrane proteins (∼250,000 subunits/cell) (Silhavy et al., 2010). Using this as a standard, densitometric analysis of protein profiles from outer membranes of *E. coli* B44 (the native source of our Wzi) suggests that there are ∼8,000 copies of Wzi/cell (I.L.M. and C.W., unpublished data). It is difficult to assess consistency using our model, because the number of CPS chains per cell is unknown and the amount of Wzi required would vary depending on the number of chains that might aggregate around a single nucleation point. Our model does not preclude the possibility that other cell-surface components play a role alongside Wzi. The model allows for the possibility that once nucleation of the polymer occurs, the protein may no longer be required. Lacking functional Wzi, the polysaccharide is synthesized and translocated but does not form a capsule; instead it is secreted. It has been demonstrated that nanospheres of carbohydrate polymers can self-assemble around a hydrophobic shell (Dou et al., 2003; Long et al., 2012). There is an obvious parallel with our model, in which Wzi is the critical factor in the initial anchoring of polymeric carbohydrate to the hydrophobic outer membrane and thereby creates a template for capsule formation.

## Experimental Procedures

### Expression, Purification, and Crystallization of Native and Selenomethionine-Substituted Wzi-His_6_

Purification and initial crystallographic studies of Wzi have been described (Bushell et al., 2010). Briefly, Wzi was expressed in *E. coli* TOP10 (F^−^, *mcrA*, Δ (*mrr-hsdRMS-mcrBC*), φ 80, *lacZ*ΔM15, Δ*lacX74*, *deoR, nupG*, *recA1*, *araD139*, Δ(*ara-leu*)7697, *galU, galK*, *repsL*(Str^r^), *endA1*) (Life Technologies), transformed with pWQ193 (Table S2). Wzi-His_6_ was solubilized using sulfobetaine 3-14 (n-tetradecyl-N,N-dimethyl-3-ammonio-1-propanesulfonate) and purified via Ni^2+^-nitrilotriacetic acid (Ni-NTA) affinity chromatography and size-exclusion chromatography into a final buffer of 20 mM Tris (pH 8.0), containing 0.1% (w/v) n-dodecyl-N,N-dimethylamine-N-oxide (LDAO). Purified protein was concentrated to ∼20 mg/ml for crystallization. Selenomethionine variant Wzi-His_6_ was expressed in the *E. coli* methionine auxotroph B834. Wzi-His_6_ was crystallized via hanging-drop vapor diffusion by mixing 0.5 μl protein solution with 0.5 μl 0.1 M MES (pH 6.0) containing 50 mM CaCl_2_ and 30% (v/v) PEG 350 MME. Rod crystals appeared in 1–2 days and grew to full size within 3–4 days. These were stored in a cryoprotectant solution of mother liquor containing 15% PEG 200 and flash frozen by immersion in liquid nitrogen. The diffraction limit of the crystals was highly variable and did not obviously correlate with the physical appearance. Wzi-His_6_ crystallized in space group *C*222, with one monomer per asymmetric unit. Data were indexed and scaled using XDS and SCALA, in XIA2 (CCP4, 1994; Kabsch, 1993; Winter, 2009). Phasing used ShelXC/D/E (Sheldrick, 2008) in hkl2map (Pape and Schneider, 2004). Automated model building was unsatisfactory, perhaps due to the high β sheet content, so manual building in Coot (Emsley et al., 2010) was used. The model was refined using TLS in Refmac5 (Murshudov et al., 2011). Full statistics are presented in Table 1; the PDB ID code is 2ynk.

### Glycan Microarray Experiments

The glycan array (Version 4.2) is maintained by the Consortium for Functional Glycomics (Emory University School of Medicine) (Heimburg-Molinaro and Rittenhouse-Olson, 2009; Smith et al., 2010). Wzi-His_6_ was tested against this array and bound protein was detected with a conjugated antibody against the C-terminal hexahistidine tag.

### Surface Plasmon Resonance Measurements

SPR measurements used BIAcore T200 instrument (GE Healthcare). NTA sensor chips, 1-ethyl-3-(3-diaminopropyl) carbodiimide hydrochloride, and N-hydroxysuccinimide were purchased from GE Healthcare. Wzi-His_6_ was immobilized on an NTA sensor chip using a modification of a published protocol (Wear and Walkinshaw, 2006; Wear et al., 2005). Following Ni^2+^ priming (60 s at 5 μl min^−1^) using buffer A (10 mM HEPES [pH 7.5] containing 150 mM NaCl, 0.05% P20) containing 500 μM NiSO_4_ and 50 μM EDTA, dextran surface carboxylate groups were activated by injection of 20 μl of 0.2 M 1-ethyl-3-(3-diaminopropyl) carbodiimide hydrochloride; 50 mM N-hydroxysuccinimide at 5 μl.min-1. Protein (between 10 nM and 100 nM in buffer A containing 50 mM EDTA) was captured and covalently stabilized on the surface to between 100 and 900 response units (RU) by injection at 30 μl min^−1^. Following attainment of the desired RU signal, a brief injection of buffer A containing 350 mM EDTA (30 s at 30 μl min^−1^) was used to remove noncovalently attached protein, followed by quenching of the unreacted succinimide esters by an injection of 20 μl of 1 M H_2_N(CH_2_)_2_OH (pH 8.5) at 5 μl min^−1^. The chip was cleaned by a wash with excess buffer A containing 50 μM EDTA at 100 μl min^−1^. The final levels of immobilized protein were between ∼180 and 900 RU. Single-cycle kinetic titration-binding SPR experiments (Karlsson et al., 2006) were performed at 25°C, using a 2-fold dilution series, ranging from 3.125 μM to 50 μM for K30 and 37.5 μM to 600 μM for N2, in buffer A containing 50 μM EDTA at 100 μl min^−1^ with a 30 s contact time and a 60 s dissociation time. The sensor surface was regenerated between experiments by dissociating any formed complex with buffer A containing 50 μM EDTA for at least 10 min. The apparent equilibrium dissociation constants (*K*_*d*_) were calculated from double-reference-corrected sensorgrams by global fitting of a 1:1 binding model, including a mass transport term (v.1.0; GE Healthcare).

### Construction of a Chromosomal wzi-Deletion Mutant

The *wzi*::*cat* mutant (CWG874) was constructed in *K. pneumoniae* 889/50 (serotype O1:K20). The mutant was constructed by allelic exchange using a suicide vector pWQ655, based on pRE118 (Edwards et al., 1998). Briefly, the chloramphenicol cassette from pKD3 (Datsenko and Wanner, 2000) was PCR-amplified and inserted between ∼500 bp of the upstream and downstream regions of *wzi*, which were amplified from *K. pneumoniae* 889/50 genomic DNA. (Primer sequences are given in Table S3). Conjugation was used to transfer pWQ655 from a donor strain, *E. coli* SM10 *λpir* (Edwards et al., 1998), to *K. pneumoniae* 889/50. Chromosomal insertion was confirmed by PCR.

### Mutagenesis of wzi

Wzi-His_6_, pWQ193 was mutagenized using QuikChange Site-directed mutagenesis (Stratagene/Agilent Technologies). Mutagenic primers (Sigma) were designed so that the desired regions were replaced with GGCGGTGGTGGCGGC (encoding 5 glycine residues), with the exception of pWQ660, in which two codons were replaced with GGCGGT. For a C-terminal FLAG-tagged derivative (Wzi-FLAG), standard PCR was used to amplify *wzi* from pWQ193 (minus the sequence encoding the hexahistidine tag) using a reverse primer that encoded the FLAG epitope (DYKDDDDK). DNA was amplified using Pfu Ultra DNA polymerase (Stratagene/Agilent Technologies) or KOD Hot Start DNA polymerase (Novagen/EMD Millipore). The selectable marker for these mutant Wzi-expressing plasmids was converted to kanamycin by inserting a SmaI fragment containing a nonpolar *aphA-3* gene (Ménard et al., 1993) into the *Sca*I restriction site of the β-lactamase gene. Mutations were confirmed by DNA sequencing (Advanced Analysis Centre, University of Guelph).

### Immunofluorescence Microscopy

*E. coli* TOP10 cells containing plasmid pWQ669 (expressing Wzi-FLAG) were grown overnight in lysogeny broth (LB) medium (Bertani, 1951) containing 0.2% glucose and ampicillin (100 μg/mL). These were then diluted to 1:50 in 6 ml of LB containing 0.02% arabinose and ampicillin and grown at 37°C for approximately 3 hr. Cells were fixed and labeled with antibodies as described (Clarke et al., 2004). Intact and permeabilized cells were labeled with monoclonal mouse anti-FLAG M2 antibody (Sigma) and detected with rhodamine red-X-conjugated goat anti-mouse IgG (Jackson ImmunoResearch Laboratories). Samples were visualized on a Leica DMRA2 microscope with a 100× objective lens and image processing used Openlab 4.0.4 (Improvision).

### Phenotypic Analysis of Wzi Mutants

A centrifugation-based assay was used, based on methods described previously (Cheng et al., 2010). *K. pneumoniae* 889/50 produces copious amounts of CPS and wild-type strains (intact capsules) therefore do not form a coherent cell pellet after low-speed centrifugation. Overnight bacterial cultures were diluted 1:50 in 10 ml of LB containing 0.02% arabinose and kanamycin (50 μg/ml), as required. Cultures were grown for 4–5 hr at 37°C and then diluted to 1 ml of OD_600_ = 2.0 in microcentrifuge tubes. The contents were centrifuged at 10000 × *g* for 5 min at 20°C. Cell pellets were imaged using a flat-bed scanner. SDS-PAGE and immunoblotting with anti-pentahistidine antibodies (QIAGEN) confirmed expression of mutants.

### Bioinformatics

The NCBI database of completed genomes was analyzed with blastp for potential Wzi homologs using “genomic BLAST.” Wzi from *E. coli* B44 (accession number AAN52285) was used as the query sequence, but the database was further probed using the sequences of potential Wzi homologs from the lead hits from Groups B and C (*Hahella chejuensis* KCTC2396 (YP_433621) and *Geobacter metallireducens* GS-15 (YP_384301)) from a preliminary tree. The results from these three searches were combined, duplicates were removed, and the list of hits was limited to Gram-negative bacteria. Secondary structures were predicted with JPRED (Cole et al., 2008). Hits were removed if they did not possess three to four α helices followed by a predominantly β strand structure. Sequences of the Wzi proteins studied here (*E. coli* B44 and *K. pneumoniae* 889/50 [BAF47011]) were added to the remaining 139 hits. A phylogenetic tree was constructed using CLC Main Workbench v5.1 (CLC bio, Denmark) using the Neighbor Joining method.

### Capsular Polysaccharide Purification

Capsular polysaccharide was purified from *Klebsiella pneumoniae* 889/50 based on the methods described in Johnson and Perry (1976) with the following modifications. Cells were obtained by scraping bacterial lawns from four large (24 cm × 38 cm) sheets of LB-agar. Total polysaccharides (CPS and LPS) were extracted in hot aqueous phenol and the retained aqueous fraction was dialyzed to remove residual phenol. LPS was removed as a pellet following ultracentrifugation of the dialyzed material. The capsular-polysaccharide-containing supernatant was lyophilized. The monomeric K30 tetrasaccharides were a gift (B. Davis and L. Kong, personal communication).

## Figures and Tables

**Table 1:** Crystallography

	Wzi
**Data collection**	

Wavelength (Å)	0.97862
Space group	*C* 222

**Cell dimensions**	

*a*, *b*, *c* (Å)	136.7, 152.8, 95.0
α, β, γ (°)	90, 90, 90
Resolution (Å)	59–2.64 (2.74–2.64)^a^
*R*_merge_	7.9 (75.2)
*R*_*meas*_ (I)	10.1 (87.7)^b^
*R*_*meas*_ (I +/−)	8.8 (87.6)
*R*_*p.i.m*_. (I)	3.0 (32.0)^c^
*R*_*p.i.m*_. (I +/−)	3.6 (44.4)
Mean *I*/σ*I*	16.9 (2.9)
Completeness (%)	99.2 (98.8)
Multiplicity	10.0 (7.4)

**Refinement**	

Resolution (Å)	59–2.64
No. reflections	27740
*R*_work_/*R*_free_	24.5/27.9

**No. atoms**	

Protein	3476
Ligand/ion	162
Water	14

***B*-factors**	

Protein	58
Ligand/ion	68
Water	46

**Rmsds**	

Bond lengths (Å)	0.007
Bond angles (°)	1.16

**Ramachandran Plot**	

Ramachandran favored	411/438 residues, 93.8%
Ramachandran allowed	436/438 residues, 99.5%

a Highest-resolution shell.

b ${\text{R}}_{\text{meas}}=\left(\sum hkl\sqrt{\frac{n}{n-1}}\sum_{j-1}^{n}Ihkl,\;j-Ihkl\right)/\left(\sum_{hkl}\sum_{j}Ihkl,\;j\right).$

c ${\text{R}}_{\text{p.i.m}}=\left(\sum hkl\sqrt{\frac{1}{n-1}}\sum_{j=1}^{n}Ihkl,\;j-Ihkl\right)/\left(\sum_{hkl}\sum_{j}Ihkl,\;j\right).$
