# Supplementary material for: Wzi Is an Outer Membrane Lectin that Underpins Group 1 Capsule Assembly in Escherichia coli
Source: Structure. 2013 May 7;21(5):844–53. doi: 10.1016/j.str.2013.03.010 (PMC3791409; doi:10.1016/j.str.2013.03.010)
Supplement: Document S1. Tables S1–S4 and Figures S1–S5 [file mmc1.pdf]

Structure, Volume *21*

Supplemental Information

Wzi Is an Outer Membrane Lectin

that Underpins Group 1 Capsule Assembly

in *Escherichia coli*

Simon R. Bushell, Iain L. Mainprize, Martin A. Wear, Hubing Lou, Chris Whitfield, and James H Naismith

Inventory of Supplemental Information

Table S1. Microarray Glycans that Display Putative Binding to Wzi, Related to Figure 3
Table S2. List of Plasmids Used in this Study, Related to Figure 2
Table S3. List of Oligonucleotide Primers, Related to Figure 2
Table S4. Putative Wzi Homolog, Related to Figure 4
Figure S1. Topology Diagram of Wzi, Related to Figure 1
Figure S2. Immunofluorescence Microscopy to Determine the Topology of Wzi, Related to Figure 1
Figure S3. SPR Sensorgrams for Non-K30-binding Wzi Mutants, Related to Figure 3
Figure S4. Comparison of Putative 'Notch' Regions in Wzi and FadL, Related to Figure 1
Figure S5. Lack of Evidence for Multivalent, Related to Figure 3

Table S1, related to Figure 3 - ***Microarray glycans that display putative binding to Wzi***

Glycan Structure	Average RFU	Standard Deviation
GlcNAcβ1-3(GlcNAcβ1-4)(GlcNAcβ1-6)GlcNAc-Sp8	1160	98
GlcNAcα1-4Galβ1-4GlcNAcβ1-3Galβ1-4(Fucα1-3)GlcNAcβ1-3Galβ1-4(Fucα1-3)GlcNAcβ-Sp0	1106	19
Neu5Acα2-3Galβ1-4GlcNAcβ1-2Manα-Sp0	831	271
Neu5Acα2-3Galβ1-4(Fucα1-3)GlcNAcβ1-3GalNAcα-Sp14	751	76
6-H_2PO_3Manα-Sp8	680	171
Fucα1-2Galβ1-4(Fucα1-3)GlcNAcβ1-3GalNAcα-Sp14	469	39
Neu5Acα2-6Galβ1-4GlcNAcβ1-6GalNAcα-Sp14	378	89
[3OSO3]Galβ1-4(Fucα1-3)[6OSO3]GlcNAc-Sp8	335	44
[3OSO3]Galβ1-4(Fucα1-3)[6OSO3]Glc-Sp0	316	49
[6OSO3]Galβ1-4[6OSO3]GlcNAcβ-Sp0	311	74

Table S2 related to Figure 2 - ***List of plasmids used in this study***

Plasmid	Description	Ref.
pWQ193	pBAD24-derivative encoding C-terminal hexa-histidine-tagged Wzi from *E.coli* B44 (Wzi-His_6); Ap^R	Rahn et al., 2003
pWQ655	pRE118-derivative containing *wzi*Kp region interrupted with *cat* cassette from pKD3[74]; Cm^R, Km^R	This study
pWQ656	pWQ193 derivative, encoding WziΔL3 with residues 171-193 replaced with 5 glycines, *aphA-3* gene inserted into ScaI of beta-lactamase gene; Km^R	This study
pWQ657	Like pWQ656 encoding WziΔL7 with residues 355-390 replaced with 5 glycine residues; Km^R	This study
pWQ658	Like pWQ656, but encoding WziΔL8 with residues 417-430 replaced with 5 glycine residues; Km^R	This study
pWQ659	Like pWQ656, but encoding WziΔL6 with residues 319-329 replaced with 5 glycine residues; Km^R	This study
pWQ660	Like pWQ656, but encoding WziΔT2 with residues 162-163 replaced with 2 glycine residues; Km^R	This study
pWQ661	Like pWQ656, but encoding WziΔT3 with residues 200-215 replaced with 5 glycine residues; Km^R	This study
pWQ662	Like pWQ656, but encoding WziΔT5 with residues 302-309 replaced with 5 glycine residues; Km^R	This study
pWQ663	Like pWQ656, but encoding WziΔH1 with residues 29-43 replaced with 5 glycine residues; Km^R	This study
pWQ664	Like pWQ656, but encoding WziΔH2 with residues 57-65 replaced with 5 glycine residues; Km^R	This study
pWQ665	Like pWQ656, but encoding WziΔH3 with residues 72-89 replaced with 5 glycine residues; Km^R	This study
pWQ666	Like pWQ656, but encoding WziΔH1A with residues 31-35 replaced with 5 glycine residues; Km^R	This study
pWQ667	Like pWQ656, but encoding WziΔH1B with residues 35-39 replaced with 5 glycine residues; Km^R	This study
pWQ668	Like pWQ656, but encoding WziΔH1C with residues 39-43 replaced with 5 glycine residues; Km^R	This study
pWQ669	Like pWQ656, but encoding WziFLAG with residues hexa-histidine tag replaced with FLAG tag; Km^R	This study

Table S3 related to Figure 2 – *List of oligonucleotide primers*

Primer	**Sequence (5'->3')**	**Applications**
ILM082	GATCCTCGAGGCTAGCCAGATATTCGGTAACAACACTCCC	Amplify upstream *wziKp* region; XhoI
ILM111	GATCGGATCCAGTTTATAAGGCTGAATGCTGAAAATAGG	BamHI
ILM112	GATCGAATTCTGTACAAGATCCATTTTCAGCCCC	Amplify downstream *wziKp* region; EcoRI
ILM083	GATCGAGCTCGTCAGAATTTACCCAGTTACCCG	SacI
ILM113	GATCGAATTCGTGTAGGCTGGAGCTGCTTC	Amplify *cat* cassette; EcoRI
ILM114	GATCGGATCCCATATGAATATCCTCCTTAGTTCCTATTC	BamHI
ILM086	CCTGGAAGCGCATGATGGCGGTGGTGGCGGCCAACTCGTTGCCGGTAAGGTTG	replace Loop L7 with 5xGly
ILM087	TTACCGGCAACGAGTTGGCCGCCACCACCGCCATCATGCGCTTCCAGGTACCAG	
ILM090	TTAGTATACGCGAAGGTCGGCGGTGGTGGCGGCGATACTCTTAAAGGTATACAGCTTGG	replace Loop L8 with 5xGly
ILM091	TGTATACCTTTAAGAGTATCGCCGCCACCACCGCCGACCTTCGCGTATACTAAGCGT	
ILM094	GGGCAGATGATCGGCGGCGGTGGTGGCGGCATGTTCCTCGGCGGGATTGAAG	replace Loop L6 with 5xGly
ILM095	CCCGCCGAGGAACATGCCGCCACCACCGCCGCCGATCATCTGCCCGTAGAA	
ILM096	GGCTCTCTTTTGGTCAGGGCGGTGGTGGCGGCGGTTTCCTGATGCAGCGAGC	replace Loop L3 with 5xGly
ILM097	GCTGCATCAGGAAACCGCCGCCACCACCGCCCTGACCAAAAGAGAGCCACTGG	
ILM098	GTTTCCTGATGCAGCGAGGCGGTGGTGGCGGCTGGCAGTACCAGATATCCGCC	replace Loop T3 with 5xGly
ILM099	GATATCTGGTACTGCCAGCCGCCACCACCGCCTCGCTGCATCAGGAAACCGG	
ILM100	GGTTTTGACTTTAAGTTCAAAGGCGGTGGTGGCGGCGTGAGCTTCTACGGGCAGATG	replace Loop T5 with 5xGly
ILM101	CCCGTAGAAGCTCACGCCGCCACCACCGCCTTTGAACTTAAAGTCAAAACCGGCCAA	
ILM102	GGCGCGGTGAAGTTCGGCGGTCAGTGGCTCTCTTTTGGTCAGG	replace Loop T2 with 2xGly
ILM103	CCAAAAGAGAGCCACTGACCGCCGAACTTCACCGCGCCGTAG	
ILM117	GCCGGGTTAGTGGTAGGCGGTGGTGGCGGCGGGGTCATCCATCTGAGC	replace Helix H1 with 5xGly
ILM118	TCAGATGGATGACCCCGCCGCCACCACCGCCTACCACTAACCCGGCCGC	
ILM119	GTGGCCGCTGAGCGGCGGTGGTGGCGGCAAGGCCAAACCTTCCTATTCTTCTG	replace Helix H2 with 5xGly
ILM120	AATAGGAAGGTTTGGCCTTGCCGCCACCACCGCCGCTCAGCGGCCACGTCG	
ILM121	AAGGCCAAACCTTCCTATGGCGGTGGTGGCGGCGCGGATTTCCGGGTCACC	replace Helix H3 with 5xGly
ILM122	GACCCGGAAATCCGCGCCGCCACCACCGCCATAGGAAGGTTTGGCCTTTTTCAGC	
ILM129	GGGTTAGTGGTAAATGACGGCGGTGGTGGCGGCGACCTGGCCTGGCTTTCCG	replace first 5 amino acids of Helix H1 with 5xGly
ILM130	AAGCCAGGCCAGGTCGCCGCCACCACCGCCGTCATTTACCACTAACCCGGCC	
ILM131	ATGACAACGACTTGCGTGGCGGTGGTGGCGGCCTTTCCGATCGCGGGGTC	replace middle 5 amino acids of Helix H1 with 5xGly
ILM132	CCCGCGATCGGAAAGGCCGCCACCACCGCCACGCAAGTCGTTGTCATTTACCAC	
ILM133	GTAACGACCTGGCCGGCGGTGGTGGCGGCGGGGTCATCCATCTGAGCC	replace last 5 amino acids of Helix H1 with 5xGly
ILM134	CAGATGGATGACCCCGCCGCCACCACCGCCGGCCAGGTCGTTACGCAAG	
ILM180	GATC**AAGCTT**TTACTTGTCGTCGTCGTCCTTGTAGTCGGATCCTAAACTAAACGGTATTT CAATCCCTGCG	Reverse primer to amplify *wzi* add FLAG tag; **HindIII**

Table S4 related to Figure 4 - *Putative Wzi homologs*

Potential Wzi homologs identified by Blastp search of NCBI bacterial genome database separated into Groups A, B, and C as determined by phylogenetic tree analysis (see Figure 4).

Organism/Strain	Accession #	Protein size (aa)
**Group A**		
Acinetobacter baumannii 6013150	ZP_08434908	502
Acinetobacter baumannii 6014059	ZP_08440827	501
Acinetobacter baumannii AB900	ZP_04662281	485
Acinetobacter baumannii ACICU	YP_001845619	480
Acinetobacter baumannii ATCC 17978	YP_001084033	480
Acinetobacter baumannii AYE	YP_001714597	481
Acinetobacter baumannii SDF	YP_001707648	480
Acinetobacter calcoaceticus RUH2202	ZP_06057685	480
Acinetobacter haemolyticus ATCC 19194	ZP_06728329	494
Acinetobacter johnsonii SH046	ZP_06062104	481
Acinetobacter junii SH205	ZP_06067806	480
Acinetobacter junii SH205	ZP_06066327	461
Acinetobacter lwoffii SH145	ZP_06070054	479
Acinetobacter radioresistens SK82	ZP_05360364	484
Acinetobacter sp. ADP1	YP_044910	485
Acinetobacter sp. ATCC 27244	ZP_03821929	494
Acinetobacter sp. DR1	YP_003733129	480
Acinetobacter sp. RUH2624	ZP_05824896	480
Acinetobacter sp. SH024	ZP_06691194	484
Brenneria sp. EniD312	EHD19937	476
Enhydrobacter aerosaccus SK60	ZP_05619161	478
Enterobacter aerogenes KCTC 2190	YP_004594883	477
Escherichia coli B44	AAN52285	479
Escherichia coli E482	EGB37058	477
Escherichia coli G58-1	EGX07314	477
Escherichia coli H736	EGI11694	504
Escherichia coli HS	YP_001458866	504
Escherichia coli MS 116-1	ZP_07160629	477
Escherichia coli MS 146-1	ZP_07243452	477
Escherichia coli MS 84-1	ZP_07120298	477
Klebsiella pneumoniae 342	YP_002237505	477
Klebsiella pneumoniae 889/50	BAF47011	479
Klebsiella pneumoniae subsp. *pneumoniae* MGH 78578	YP_001336161	477
Klebsiella pneumoniae subsp. *pneumoniae* NTUH-K2044	YP_002920367	504
Klebsiella pneumoniae subsp. *rhinoscleromatis* ATCC 13884	ZP_06013341	479
Klebsiella sp. 1_1_55	ZP_06548003	504
Klebsiella sp. MS 92-3	ZP_08304229	477
Klebsiella variicola At-22	YP_003438487	477
Providencia rettgeri DSM 1131	ZP_06127575	482
Providencia stuartii ATCC 25827	ZP_02958757	479
Psychrobacter arcticus 273-4	YP_263965	492
Psychrobacter cryohalolentis K5	YP_579906	483
Psychrobacter sp. 1501(2011)	ZP_08460690	490
Psychrobacter sp. PRwf-1	YP_001279137	497
Serratia odorifera 4Rx13	ZP_06191623	478
Serratia odorifera DSM 4582	ZP_06638359	477
Serratia proteamaculans 568	YP_001480698	477
Serratia sp. AS12	YP_004502981	478
**Group B**		
Alcanivorax borkumensis SK2	YP_692624	509
Alcanivorax sp. DG881	ZP_05040828	492
Aliivibrio salmonicida LFI1238	YP_002261812	455
Alkalilimnicola ehrlichii MLHE-1	YP_743178	512
Alkalilimnicola ehrlichii MLHE-1	YP_741641	493

Alteromonadales bacterium TW-7	ZP_01611635	514
Glaciecola nitratireducens FR1064	YP_004870208	493
Hahella chejuensis KCTC 2396	YP_433621	496
Idiomarina loihiensis L2TR	YP_155443	436
Idiomarina sp. A28L	ZP_08621944	511
Kangiella koreensis DSM 16069	YP_003146104	484
Marinobacter algicola DG893	ZP_01894563	497
Marinomonas sp. MWYL1	YP_001339662	444
Pseudoalteromonas haloplanktis ANT/505	ZP_08410341	501
Pseudoalteromonas haloplanktis TAC125	YP_340820	515
Pseudoalteromonas sp. SM9913	YP_004067863	515
Pseudoalteromonas tunicata D2	ZP_01133106	482
Psychromonas ingrahamii 37	YP_942209	391
Rheinheimera sp. A13L	ZP_08571600	471
Shewanella amazonensis SB2B	YP_925993	494
Shewanella baltica OS155	YP_001048474	491
Shewanella baltica OS185	YP_001368507	491
Shewanella baltica OS223	YP_002360164	491
Shewanella benthica KT99	ZP_02157001	487
Shewanella benthica KT99	ZP_02156132	497
Shewanella denitrificans OS217	YP_561229	494
Shewanella frigidimarina NCIMB 400	YP_752589	499
Shewanella halifaxensis HAW-EB4	YP_001673746	488
Shewanella halifaxensis HAW-EB4	YP_001672371	499
Shewanella loihica PV-4	YP_001093552	488
Shewanella loihica PV-4	YP_001095816	501
Shewanella oneidensis MR-1	NP_720201	494
Shewanella pealeana ATCC 700345	YP_001501298	488
Shewanella pealeana ATCC 700345	YP_001503951	499
Shewanella piezotolerans WP3	YP_002310989	488
Shewanella piezotolerans WP3	YP_002314196	494
Shewanella putrefaciens CN-32	YP_001185392	491
Shewanella sediminis HAW-EB3	YP_001474673	487
Shewanella sediminis HAW-EB3	YP_001471857	497
Shewanella sp. ANA-3	YP_871713	490
Shewanella sp. HN-41	ZP_08564602	490
Shewanella sp. MR-4	YP_736007	490
Shewanella sp. MR-7	YP_740016	490
Shewanella sp. W3-18-1	YP_961468	491
Shewanella violacea DSS12	YP_003556266	488
Shewanella violacea DSS12	YP_003554861	498
Shewanella woodyi ATCC 51908	YP_001760087	488
Shewanella woodyi ATCC 51908	YP_001758473	501
Teredinibacter turnerae T7901	YP_003072998	453
Thermodesulfobacterium sp. OPB45	YP_004627398	472
Verrucomicrobiae bacterium DG1235	ZP_05060372	468
Verrucomicrobiae bacterium DG1235	ZP_05055994	505
Vibrio coralliilyticus ATCC BAA-450	ZP_05888471	422
Vibrio nigripulchritudo ATCC 27043	ZP_08734212	406
Vibrio tubiashii ATCC 19109	ZP_08736967	414
**Group C**		
Acidobacterium capsulatum ATCC 51196	YP_002753497	575
alpha proteobacterium BAL199	ZP_02188264	459
Alteromonadales bacterium TW-7	ZP_01612085	480
Candidatus Koribacter versatilis Ellin345	YP_592387	582
Candidatus Koribacter versatilis Ellin345	YP_589912	581
Candidatus Koribacter versatilis Ellin345	YP_592882	561
Candidatus Nitrospira defluvii	YP_003796579	583

Chloroherpeton thalassium ATCC 35110	YP_001997054	518
Colwellia psychrerythraea 34H	YP_270854	448
Ferrimonas balearica DSM 9799	YP_003912367	495
Flexistipes sinusarabici DSM 4947	YP_004602982	504
Geobacter bemidjiensis Bem	YP_002138450	470
Geobacter bemidjiensis Bem	YP_002138449	543
Geobacter daltonii FRC-32	YP_002537595	557
Geobacter lovleyi SZ	YP_001951750	542
Geobacter metallireducens GS-15	YP_006720308	559
Geobacter sp. M18	YP_004200264	516
Geobacter sp. M18	YP_004198211	547
Geobacter sp. M21	YP_003022377	464
Geobacter sp. M21	YP_003022378	543
Geobacter sulfurreducens PCA	NP_952895	549
Geobacter uraniireducens Rf4	YP_001231910	560
Glaciecola sp. 4H-3-7+YE-5	YP_004432296	467
Granulicella tundricola MP5ACTX9	YP_004219643	596
Pectobacterium atrosepticum SCRI1043	YP_049044	737
Pelobacter propionicus DSM 2379	YP_903034	540
Pseudoalteromonas atlantica T6c	YP_659640	467
Pseudoalteromonas haloplanktis ANT/505	ZP_08410186	479
Pseudoalteromonas tunicata D2	ZP_01133099	470
Saccharophagus degradans 2-40	YP_529205	512
Syntrophobacter fumaroxidans MPOB	YP_844908	580
Syntrophobacter fumaroxidans MPOB	YP_846310	592
Teredinibacter turnerae T7901	YP_003072323	478
Terriglobus saanensis SP1PR4	YP_004182970	606
Terriglobus saanensis SP1PR4	YP_004183382	559
Terriglobus saanensis SP1PR4	YP_004181319	632

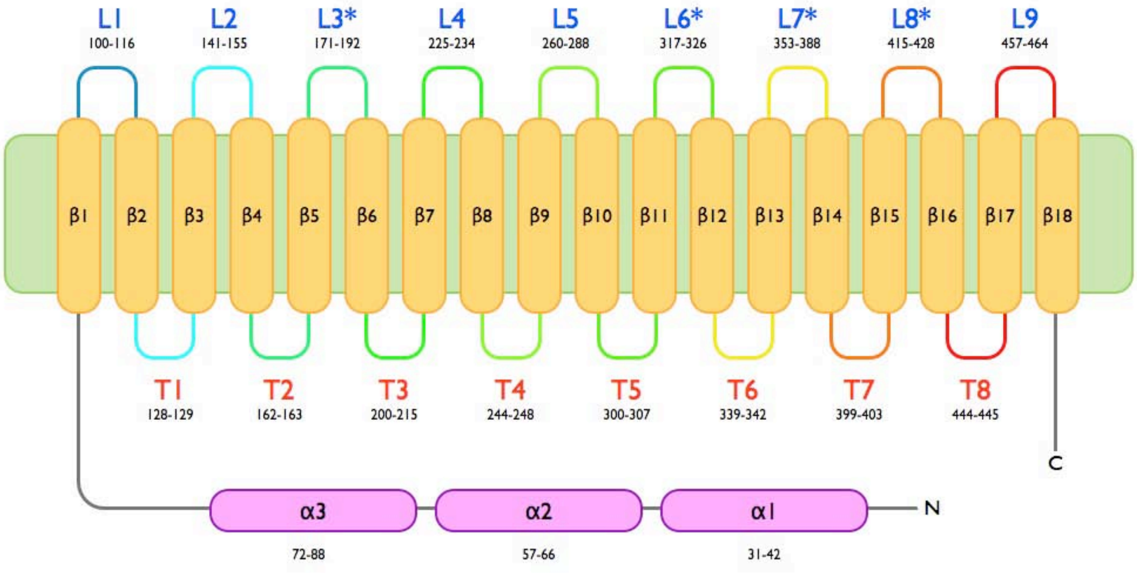

Fig S1

Supplementary Figure S1 - ***Topology diagram of Wzi***

Diagram showing the location of secondary structure features in relation to the outer membrane. Extracellular loops and periplasmic loop regions are coloured identically to the colour scheme used in Figure 1C. Extracellular loops labelled with an asterisk (*) indicate loops that were deleted for testing K30 binding via SPR. Numbers below each label indicate residue number where the feature begins and ends.

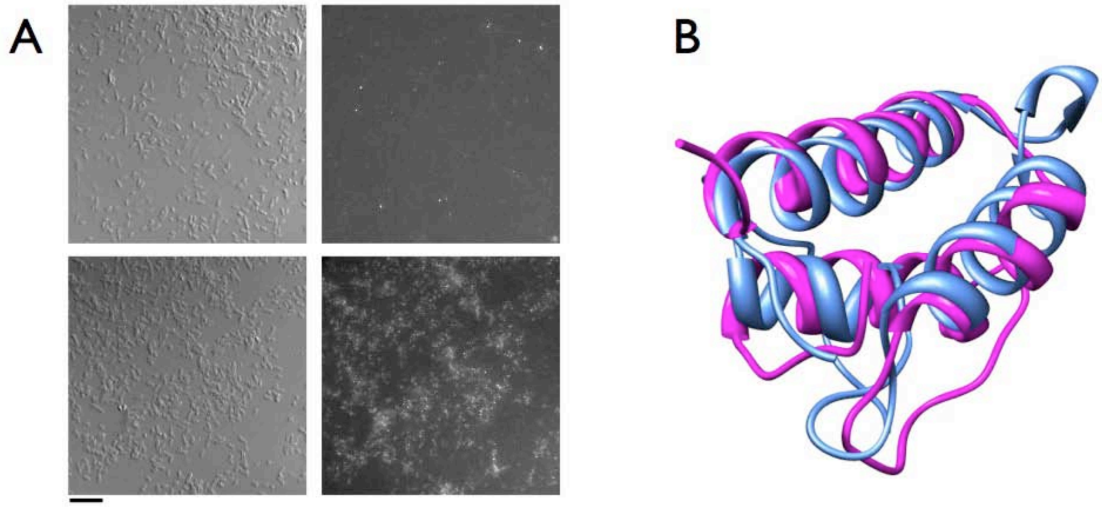

Fig S2

Supplementary Figure S2 related to Figure 1 - ***Immunofluorescence microscopy to determine the topology of Wzi***

(A) Intact (*top*) and permeabilized (*bottom*) TOP10 cells expressing WziFLAG. The FLAG-epitope tag was detected only when the cells were permeabilized, confirming the expected periplasmic location. A differential interference contrast (*left*) and a wide-field fluorescence image (*right*) are shown for each condition. Scale bar = 10 μm. **(B)** Overlay of the periplasmic helical bundle of Wzi (magenta) with Tim16 (blue) (PDB id: 2GUZ, Chain A), as calculated using *PDBeFold*.

A

WziΔL2

B

WziΔL6

C

WziΔL7

Fig S3

Supplementary Figure S3 related to Figure 3 - ***SPR sensorgrams for non-K30-binding Wzi mutants***

Sensorgrams showing the failure of **(A)** Wzi ΔL2 **(B)** Wzi ΔL6 and **(C)** Wzi ΔL7 to bind to titrated polymeric K30.

Fig S4

Supplementary Figure S4 related to Figure 1 - ***Comparison of putative 'notch' regions in Wzi and FadL.***

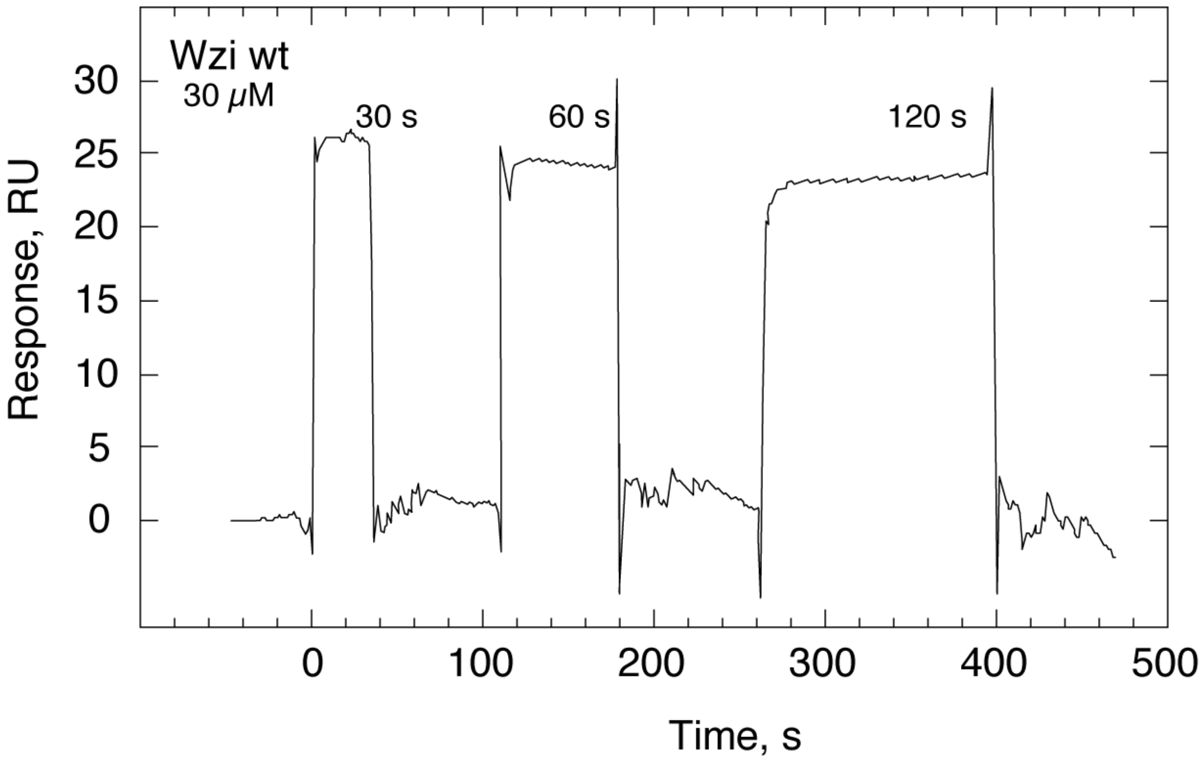

Figure S5

Supplementary Figure S5 related to Figure 3 - ***Lack of evidence for multivalent effects*****.** 30 µM K30 was injected (30 µl.min-1) over a surface (840 RU) of covalently capture/stabilized wild-type Wzi protein with varying steady-state contact times; 30 s, 60 s and 120 s. Baseline was achieved prior to re-injection of the analyte. No significant difference in the association or dissociation profiles was observed, suggesting that any avidity/multi-valency effects were minimal.
